# Mapping the stereotyped behaviour of freely moving fruit flies

**DOI:** 10.1098/rsif.2014.0672

**Published:** 2014-10-06

**Authors:** Gordon J. Berman, Daniel M. Choi, William Bialek, Joshua W. Shaevitz

**Affiliations:** 1 Joseph Henry Laboratories of Physics and Lewis-Sigler Institute for Integrative Genomics, Princeton University, Princeton, NJ 08544, USA; 2 Department of Molecular Biology, Princeton University, Princeton, NJ 08544, USA

**Keywords:** behaviour, stereotypy, unsupervised learning, *Drosophila*, phase reconstruction

## Abstract

A frequent assumption in behavioural science is that most of an animal's activities can be described in terms of a small set of stereotyped motifs. Here, we introduce a method for mapping an animal's actions, relying only upon the underlying structure of postural movement data to organize and classify behaviours. Applying this method to the ground-based behaviour of the fruit fly, *Drosophila melanogaster*, we find that flies perform stereotyped actions roughly 50% of the time, discovering over 100 distinguishable, stereotyped behavioural states. These include multiple modes of locomotion and grooming. We use the resulting measurements as the basis for identifying subtle sex-specific behavioural differences and revealing the low-dimensional nature of animal motions.

## 1. Introduction

The concept of stereotypy—that an organism's behaviours can be decomposed into discrete, reproducible elements—has influenced the study of ethology, behavioural genetics and neuroscience for decades [1,2]. Animals possess the ability to move in a vast continuum of ways, theoretically constrained only by the biomechanical limits of their own morphology. Despite this, the range of behavioural actions typically performed by an animal is thought to be much smaller, constructed largely of stereotyped actions that are consistent across time, individuals and, in some cases, even species [3,4]. A discrete behavioural repertoire can potentially arise via a number of mechanisms, including mechanical limits of gait control, habit formation and selective pressure to generate robust or optimal actions. In many instances, the search for an individual behavioural neural circuit or gene begins with the assumption that a particular action of interest is stereotyped across time and individuals [5,6].

Despite the centrality of this concept, with few exceptions [7–11], the existence of stereotypy has not been probed experimentally. This is largely due to the lack of a comprehensive and compelling mathematical framework for behavioural analysis. Here, we introduce a new method for quantifying postural dynamics that retains an animal's full behavioural complexity, using the fruit fly *Drosophila melanogaster* as a model organism to discover and map stereotyped motions.

Most prior methods for quantifying animal behaviour lie in one of two regimes. One of these is the use of coarse metrics such as a gross activity level (e.g. mean velocity or number of times the organism crosses a barrier) or counting the relative frequencies of particular events engrained into the experimental set-up (e.g. turning left or right in a maze). While this approach allows for high-throughput analysis of various organisms, strains and species, only the most gross aspects of behaviour can be ascertained, potentially overlooking the often subtle effects of the manipulations of interest that are only apparent at a finer descriptive level. The other common approach for behavioural quantification is to use a set of user-defined behavioural categories. These categories, such as walking, grooming or fighting, are codified heuristically and scored either by hand or, more recently, via supervised machine-learning techniques [12–16]. While the latter approach allows for higher throughput and more consistent labelling, it remains prone to human bias and anthropomorphism and often precludes objective comparisons between datasets due to the reliance on subjective definitions of behaviour. Furthermore, these analyses assume, *a priori*, that stereotyped classes of behaviour exist without first showing, from the data, that an organism's actions can be meaningfully categorized in a discrete manner.

Ideally, a behavioural description should manifest itself directly from the data, based upon clearly stated assumptions, each with testable consequences. The basis of our approach is to view behaviour as a trajectory through a high-dimensional space of postural dynamics. In this space, discrete behaviours correspond to epochs in which the trajectory exhibits pauses, corresponding to a temporally extended bout of a particular set of motions. Epochs that pause near particular, repeatable positions represent stereotyped behaviours. Moreover, moments in time in which the trajectory is not stationary, but instead moves rapidly, correspond to non-stereotyped actions.

In this paper, we construct a behavioural space for freely moving fruit flies. We observe that the flies exhibit approximately 100 stereotyped behaviours that are interspersed with frequent bouts of non-stereotyped behaviours. These stereotyped behaviours manifest themselves as distinguishable peaks in the behavioural space and correspond to recognizably distinct behaviours such as walking, running, head grooming, wing grooming, etc. Using this framework, we begin to address biological questions about the underlying postural dynamics that generate behaviour, opening the door for a wide range of other inquiries into the dynamics, neurobiology and evolution of behaviour.

## 2. Experiments

**Figure 1.**
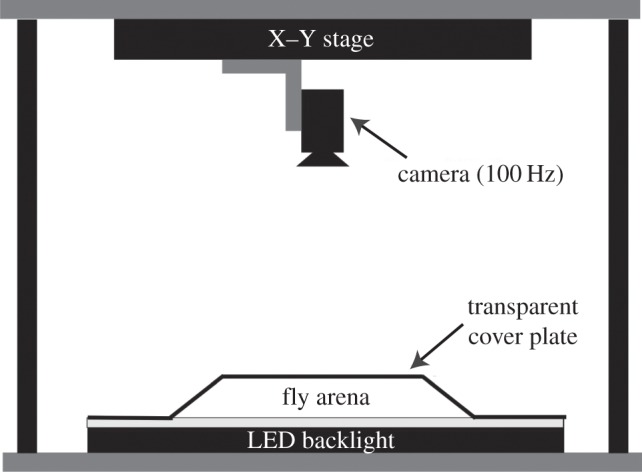
Schematic of the imaging apparatus.

We probed the spontaneous behaviours of ground-based flies (*D. melanogaster*) in a largely featureless circular arena (figure 1). Under these conditions, flies display a multitude of complex, non-aerial behaviours such as locomotion and grooming, typically involving multiple parts of their bodies. To capture dynamic rearrangements of the fly's posture, we recorded videos of individual behaving animals with sufficient spatio-temporal resolution to resolve moving body parts such as the legs, wings and proboscis.

We designed our arena based on previous work which showed that a thin chamber with gently sloping sides prevents flies from flying, jumping and climbing the walls [17]. To keep the flies in the focal plane of our camera, we inverted the previous design. Our arena consists of a custom-made vacuum-formed, clear PETG plastic dome 100 mm in diameter and 2 mm in height with sloping sides at the edge clamped to a flat glass plate. The edges of the plastic cover are sloped to prevent the flies from being occluded and to limit their ability to climb upside-down on the cover. The underside of the dome is coated with a repellent silane compound (heptane and 1,7-dichloro-1,1,3,3,5,5,7,7-octamethyltetrasiloxane) to prevent the flies from adhering to the surface. In practice, we find that this set-up results in no bouts of upside-down walking.

Over the course of these experiments, we studied the behaviour of 59 male and 51 female *D. melanogaster* (Oregon-R strain). Each animal was imaged using a high-speed camera (100 Hz, 1088 × 1088 pixels). A proportional–integral–derivative feedback algorithm is used to keep the moving fly inside the camera frame by controlling the position of the X–Y stage based on the camera image in real time. In each frame, we focus our analysis on a 200 × 200 pixel square containing the fly. We imaged each of the flies for 1 h, yielding 3.6 × 10^5^ movie frames per individual, or approximately 4 × 10^7^ frames in total. All aspects of the instrumentation are controlled by a single computer using a custom-written LabView graphical user interface.

Each of these flies was isolated within 4 h of eclosion and imaging occurred 1–14 days after that. Flies were placed into the arena via aspiration and were subsequently allowed 5 min for adaptation before data collection (electronic supplementary material, figure S1). All recording occurred between the hours of 9.00 and 13.00, thus reducing the effect of circadian rhythms, and the temperature during all recordings was 25 ± 1°C.

## 3. Behavioural analysis

**Figure 2.**
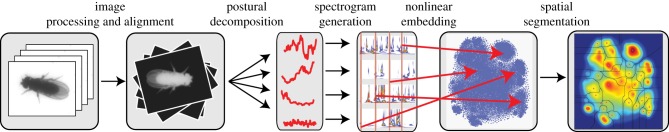
Overview of the data analysis pipeline. Raw images of the *D. melanogaster* are segmented from the background, rescaled to a reference size and then aligned, creating a stack of images in the co-moving and co-rotating frame of the fly. These images are then decomposed via PCA into a relatively low-dimensional set of time series. A Morlet wavelet transform is subsequently applied to these time series, creating a spectrogram for each postural mode separately. After normalization, each point in time is mapped into a two-dimensional plane via *t*-SNE [18]. Lastly, a watershed transform is applied to a Gaussian-smoothed density over these points, isolating individual peaks from one another.

The general framework of our analysis is described in figure 2. Images are first segmented and registered in order to isolate the fly from the background and enforce translational and rotational invariance. After this, they are decomposed into postural time series and converted into wavelet spectrograms, thus creating a spatio-temporal representation for the fly's dynamics within the images. These spectrograms are used to construct spectral feature vectors that we embed into two dimensions using *t*-distributed stochastic neighbour embedding (*t*-SNE) [18]. Lastly, we estimate the probability distribution over this two-dimensional space and identify resolvable peaks in the distribution. We confirm that sustained pauses near these peaks correspond to discrete behavioural states.

### 3.1. Image segmentation and registration

Given a sequence of images, we wish to build a spatio-temporal representation for the fly's postural dynamics. We start by isolating the fly within each frame, followed by rotational and translational registration to produce a sequence of images in the coordinate frame of the insect. Details of these procedures are listed in appendix A. In brief, we apply Canny's method for edge detection [19], morphological dilation and erosion to create a binary mask for the fly. After applying this mask, we rotationally align the images via polar cross-correlation with a template image, similar to previously developed methods [20–22]. We then use a sub-pixel cross-correlation to translationally align the images [23]. Lastly, every image is re-sized so that, on average, each fly's body covers the same number of pixels. An example segmentation and alignment is shown in the electronic supplementary material, movie S1.

### 3.2. Postural decomposition

As the fly body is made up of relatively inflexible segments connected by mobile joints, the number of postural degrees of freedom is relatively small when compared with the 40 000 pixels in each image. Accordingly, a natural representation for the fly's posture would be to enumerate the relative angles of each of the fly's appendages as a function of time [24–26]. Extracting these variables directly from the images, however, is prohibitively difficult due to occlusions and the complex fly limb and wing geometry.

**Figure 3.**
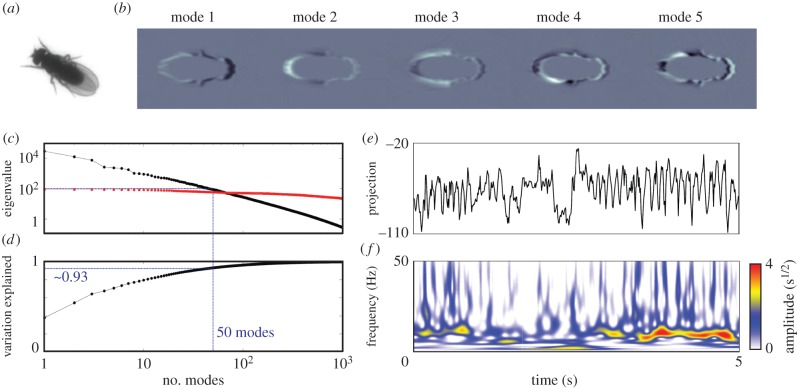
Generation of spectral feature vectors. (*a*) Raw image of a fly in the arena. (*b*) Pictorial representation of the first five postural modes, 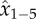, after inverse Radon transform. Black and white regions represent highlighted areas of each mode (with opposite sign). (*c*) First 1000 eigenvalues of the data matrix (black) and shuffled data (red). (*d*) Fraction of cumulative variation explained as a function of the number of modes included. (*e*) Typical time series of the projection along postural mode 6 and (*f*) its corresponding wavelet transform.

As an alternative strategy, we find that nearly all of the variance in the 4 × 10^4^ pixel images can be explained by projecting the observed pixel values onto a Euclidean space of just 50 dimensions. We apply principal component analysis (PCA) to Radon transforms of the images. PCA is a frequently used method for converting a set of correlated variables into a set of values of linearly uncorrelated eigenmodes. Results from this analysis can be described as the space spanned by the eigenvectors of the data covariance matrix, *C*, corresponding to the largest *m* eigenvalues out of the total latent dimensionality of the data. While, in general, there is no rigorous manner to choose *m*, here, we will keep all modes containing correlations larger than the finite sampling error within our dataset. According to this heuristic, we set *m* = 50 (figure 3*c*), a number of modes explaining approximately 93% of the observed variation (figure 3*d*). Details of this computation can be found in appendix B.

We refer to these directions of correlated variation as postural modes. As seen in figure 3*b*, these modes are fly-like in appearance, but do not lend themselves to intuitive interpretation. However, projecting individual images onto these axes, we can convert a movie of fly behaviour into a 50-dimensional time series,3.1
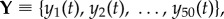
as exemplified in figure 3*e*.

### 3.3. Spectrogram generation

The instantaneous values of the postural modes do not provide a complete description of behaviour, as our definition of stereotypy is inherently dynamical. Previously published studies of behaviour have searched for motifs—repeated subsequences of finite length—within a behavioural time series [11,27]. However, this paradigm is often confounded by problems of temporal alignment and relative phasing between the component time series. Additionally, certain behaviours (for example, wing grooming in *Drosophila*) involve multiple appendages moving at different time scales, thus complicating the choice of motif length.

As an alternative to this approach, we use a spectrogram representation for the postural dynamics, measuring the power at frequency *f* associated with each postural mode, *y_k_*(*t*), in a window surrounding a moment in time, *S*(*k*, *f*; *t*). More specifically, we compute the amplitudes of the Morlet continuous wavelet transform for each postural mode [28]. Although similar to a Fourier spectrogram, wavelets possess a multi-resolution time–frequency trade-off, allowing for a more complete description of postural dynamics occurring at several time scales [29]. In particular, the Morlet wavelet is adept at isolating chirps of periodic motion, similar to what we observe in our dataset. By measuring only the amplitudes of the transform, we eliminate the need for precise temporal alignment that is required in any motif-based analysis. Details of these calculations are shown in appendix C, and an example spectrogram is displayed in figure 3*f*. For the results presented here, we look at 25 frequency channels, dyadically spaced between 1 and 50 Hz, the larger of which being the Nyquist frequency of the system.

### 3.4. Spatial embedding

*S*(*k*, *f*; *t*) comprises 25 frequency channels for each of the 50 eigenmodes, making each point in time represented by a 1250-dimensional feature vector encoding the postural dynamics. As correlations, often strong, exist between the various mode–frequency channels, we expect that the dimensionality of the manifold containing the observed values of *S*(*k*, *f*; *t*) should be vastly smaller. As such, we would like to find a low-dimensional representation that captures the important features of the dataset.

Our strategy for dimensional reduction of the feature vectors is to construct a space, *B*, such that trajectories within it pause near a repeatable position whenever a particular stereotyped behaviour is observed. This means that our embedding should minimize any local distortions. However, we do not require preservation of structure on longer length scales. Hence, we chose an embedding that reduces dimensionality by altering the distances between more distant points on the manifold.

Most common dimensionality reduction methods, including PCA, multi-dimensional scaling and Isomap, do precisely the opposite, sacrificing local verisimilitude in service of larger scale accuracy [30–32]. One method that does possess this property is *t*-SNE [18]. Like other embedding algorithms, *t*-SNE aims to take data from a high-dimensional space and embed it into a space of much smaller dimensionality, preserving some set of invariants as best as possible. For *t*-SNE, the conserved invariants are related to the Markov transition probabilities if a random walk is performed on the dataset. Specifically, we define the transition probability from time point *t_i_* to time point *t_j_*, *p_j_*_|*i*_, to be proportional to a Gaussian kernel of the distance (as of yet, undefined) between them3.2
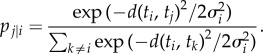


All self-transitions (i.e. *p_i_*_|*i*_) are assumed to be zero. Each of the *σ*_*i*_ are set such that all points have the same transition entropy, *H_i_* = *∑_j_p_j|i_* log *p_j|i_* = 5. This can be interpreted as restricting transitions to roughly 32 neighbours.

The *t*-SNE algorithm then embeds the data points in the smaller space while keeping the new set of transition probabilities, *q_j|i_*, as similar to the *p_j_*_|*i*_ as possible. The *q_j|i_* are defined similarly to the larger space transition probabilities, but are now, for technical reasons, proportional to a Cauchy (or Student-*t*) kernel of the points' Euclidean distances in the embedded space. This algorithm results in an embedding that minimizes local distortions [18]. If *p_j_*_|*i*_ is initially very small or zero, it will place little to no constraint on the relative positions of the two points, but if the original transition probability is large, it will factor significantly into the cost function.

This method's primary drawback, however, is its poor memory complexity scaling (∝*N*^2^). To incorporate our entire dataset into the embedding, we use an importance sampling technique to select a training set of 35 000 data points, build the space from these data, and then re-embed the remaining points into the space as best as possible (see appendix D for implementation details).

Lastly, we need to define a distance function, *d*(*t_i_*, *t_j_*), between the feature vectors. We desire this function to accurately measure how different the shapes of two mode–frequency spectra are, ignoring the overall multiplicative scaling that occurs at the beginning and the end of behavioural bouts due to the finite nature of the wavelet transform. Simply measuring the Euclidean norm between two spectra will be greatly affected by such amplitude modulations. However, because *S*(*k*, *f*; *t*) is composed of a set of wavelet amplitudes, it must therefore be positive semi-definite. As such, if we define3.3
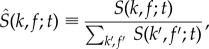


then we can treat this normalized feature vector as a probability distribution over all mode–frequency channels at a given point in time. Hence, a reasonable distance function is the Kullback–Leibler (KL) divergence [33] between two feature vectors3.4
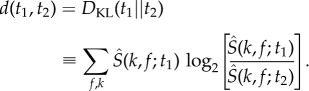


## 4. Results

### 4.1. Embedded space dynamics

**Figure 4.**
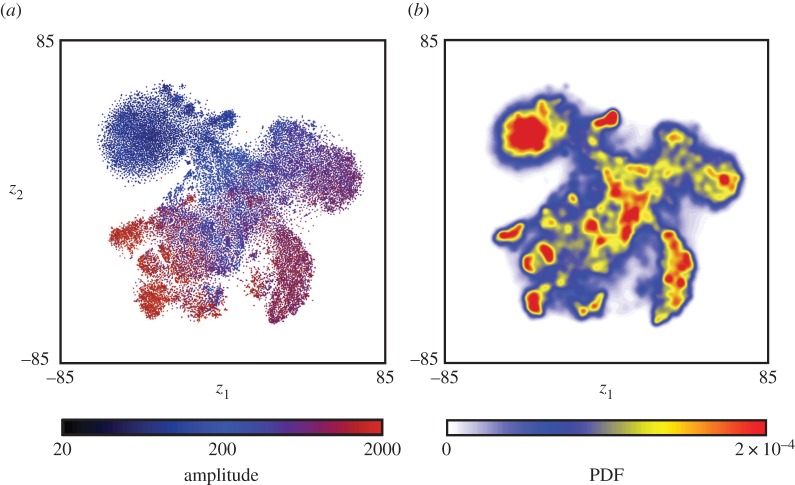
Embedding of feature vectors. (*a*) Training set points embedded into two dimensions via *t*-SNE. Colour coding is proportional to the logarithm of the normalization factor ∑*_k_*_,*f*_ *S*(*k*, *f*; *t*). (*b*) Probability density function (PDF) generated from embedding all data points and convolving with a Gaussian (*σ* = 1.5).

Figure 4 shows the embedding of our spectral feature vectors into two dimensions, the space (*z*_1_, *z*_2_), for all of the 59 individual male flies. We first note that nearby points have similar power (∑*_k_*_,*f*_ *S*(*k*, *f*; *t*)), even though the embedding algorithm normalizes-out variations in the total power of the postural motions. Embedding the same data into three dimensions yields a very similar structure with less than 2% reduction of the embedding cost function (equation (D 1); electronic supplementary material, figure S3).

We generated an estimate of the probability density, *b*(**z**), by convolving each point in the embedded map with a Gaussian of relatively small width (*σ* = 1.5, figure 4*b*). Far from being uniformly distributed across this space, *b*(**z**) contains a large number of resolved local maxima. The locations of these peaks provide a potential representation for the stereotyped behaviours that the flies perform. As expected, we find that individuals display significantly less intra- than inter-individual variation when their behavioural maps are compared (electronic supplementary material, figure S4).

**Figure 5.**
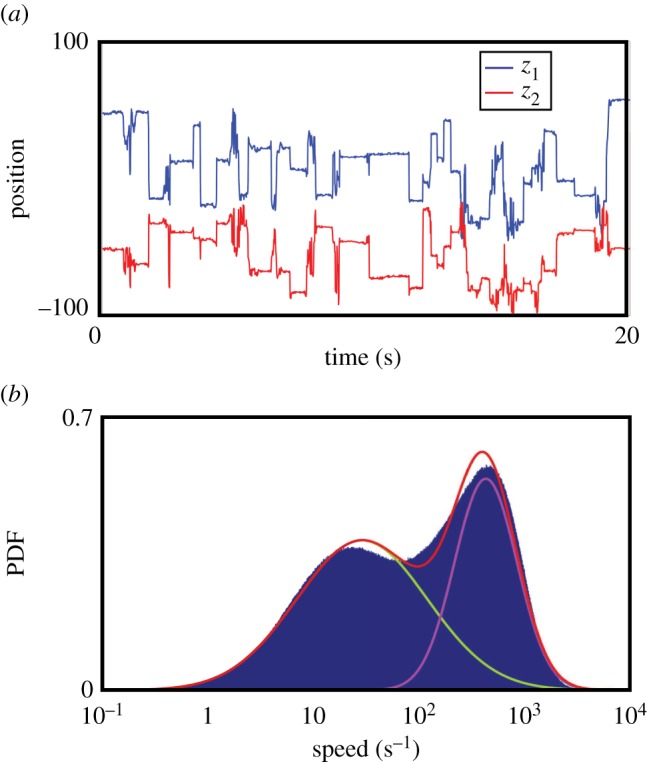
Dynamics within behavioural space. (*a*) Typical trajectory segment through behavioural space, *z*_1_(*t*) (blue) and *z*_2_(*t*) (red). (*b*) Histogram of velocities in the embedded space fitted to a two-component log-Gaussian mixture model. The blue bar chart represents the measured probability distribution, the red line is the fitted model, and the cyan and green lines are the mixture components of the fitted model.

**Figure 6.**
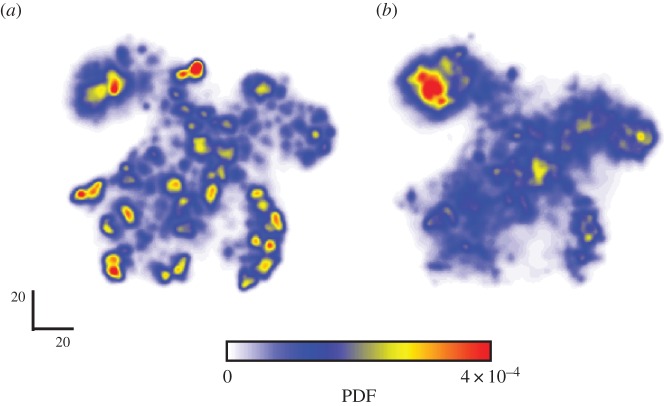
Concentration of behavioural space during stereotyped movements. Comparison between the densities generated during (*a*) stereotyped and (*b*) non-stereotyped epochs.

This space not only contains peaks, but the trajectory through it also pauses at repeatable locations. Through numerical differentiation of *z*_1_(*t*) and *z*_2_(*t*), we observe a two-state ‘pause–move’ pattern of dynamics. Typical time traces of *z*_1_(*t*) and *z*_2_(*t*) show this type of trajectory, with long stationary periods interspersed by quick bouts of movement (figure 5*a*). More quantitatively, we find that the distribution of velocities within the embedded space is well represented by a two-component lognormal mixture model in which the two peaks are separated by almost two orders of magnitude (figure 5*b*). The distribution of points in the low-velocity case (approx. 45% of all time points) is highly localized with distinguishable peaks (figure 6). The high-velocity points, in contrast, are more uniformly distributed.

### 4.2. Behavioural states

**Figure 7.**
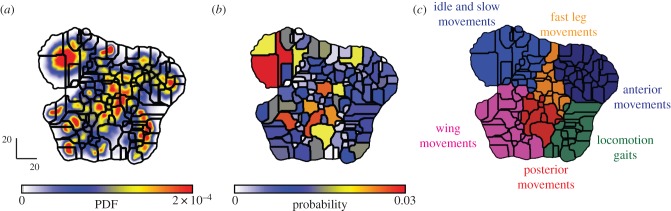
Segmentation into behavioural regions. (*a*) Boundary lines obtained from performing a watershed transform on the PDF from figure 4*b*. (*b*) Integrated probabilities within each of the regions. (*c*) The organization of behavioural space into regions of similar movement types. Definition of regions is performed through visual assessment of movies.

**Figure 8.**
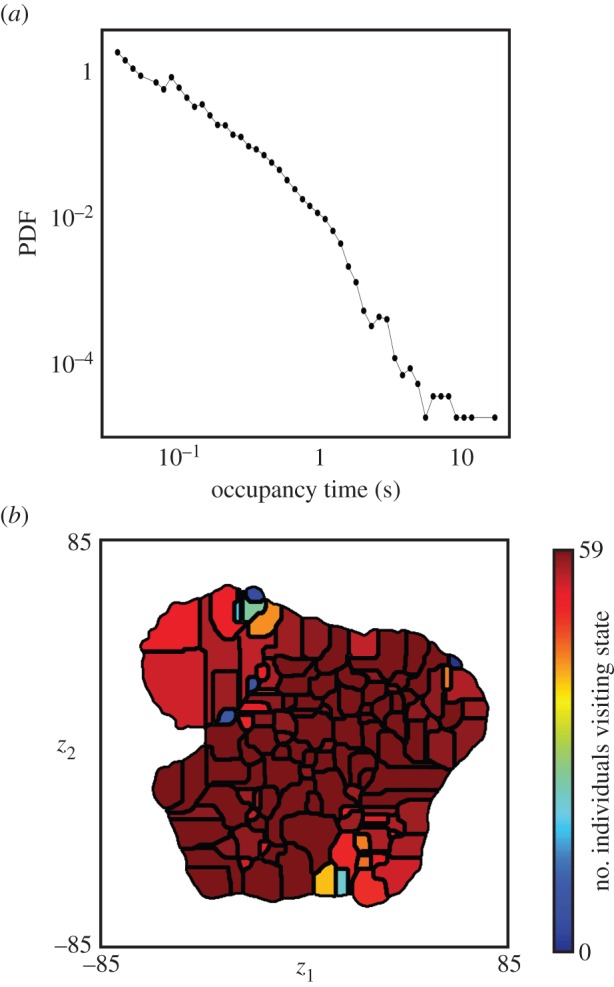
Behavioural state dynamics. (*a*) A distribution of occupancy times in all behaviours. (*b*) Number of individuals (out of 59 possible) that visit each behaviour at some point during observation.

The embedded space comprises peaks surrounded by valleys. Finding connected areas in the *z*_1_,*z*_2_ plane such that climbing up the gradient of probability density always leads to the same local maximum, often referred to as a watershed transform [34], we delineate 122 regions of the embedded space. Each of these contains a single local maximum of probability density (figure 7*a*). When the trajectory, **z**(*t*), pauses at one of these peaks, we find that each of these epochs corresponds to the fly performing a particular stereotyped behaviour. These pauses last anywhere from 0.05 s up to nearly 25 s (figure 8*a*).

Observing segments of the original movies corresponding to pauses in one of the regions, we consistently observe the flies performing a distinct action that corresponds to a recognizable behaviour when viewed by eye (electronic supplementary material, movies S2–S11). Many of the movements we detect are similar to familiar, intuitively defined behavioural classifications such as walking, running, front leg grooming and proboscis extension, but, here, the segmentation of the movies into behavioural categories has emerged from the data themselves, not through *a priori* definitions. Moreover, we see that nearby regions of our behavioural space correspond to similar, yet distinct, behaviours (figure 7*c*).

**Figure 9.**
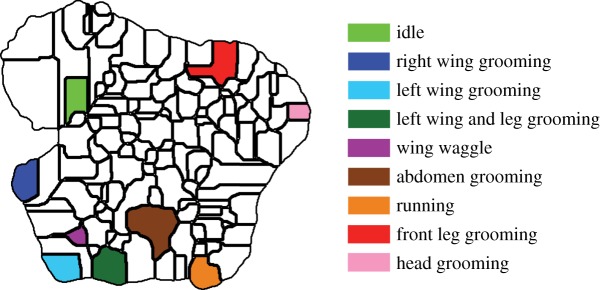
Behavioural space peaks correspond to specific stereotyped behaviours. Selected regions within behavioural space are shown and are labelled via the colour-coded legend on the right. Instances of dwells within each of these regions can be seen in the electronic supplementary material, movies S3–S11. The examples displayed in these movies are randomly selected and contain clips from many different flies, showing that the behavioural space provides a representation of behaviour that is consistent across individuals.

This classification is consistent across individuals (figures 8 and 9; electronic supplementary material, movies S3–S11). The vast majority of these regions are visited by almost all of the flies at some point (figure 8*b*). One hundred and four of the 122 regions were visited by over 50 (of 59 total) flies, and the remaining behaviours were all low-probability events, containing, in total, less than 3% of the overall activity.

### 4.3. Behavioural states as periodic orbits

**Figure 10.**
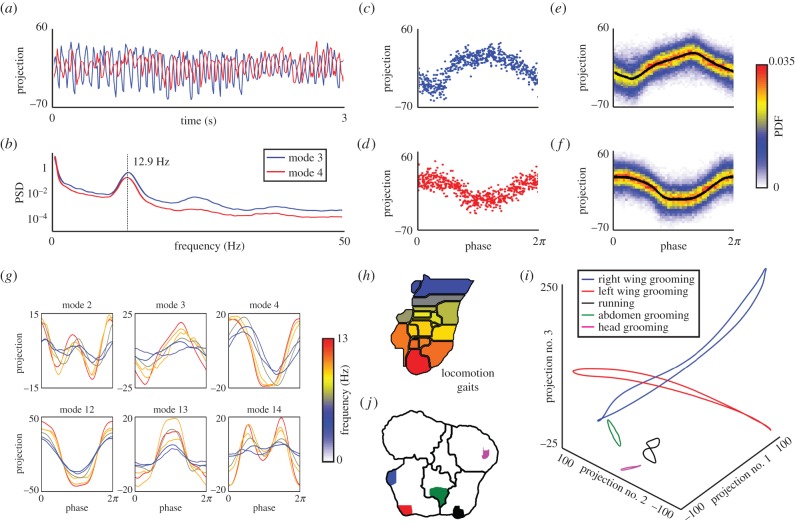
Periodic dynamics within behavioural states. (*a*) Periodic oscillations in the third and fourth postural eigenmodes during a typical high-frequency running sequence. (*b*) Average power spectral density (PSD) for all instances of this behaviour (the bottom-most region in (*h*)). Panels (*c*) and (*d*) represent phase reconstruction of the data in (*a*) for modes 3 and 4, respectively. Panels (*e*) and (*f*) represent probability densities of projections along the third and fourth modes, respectively, for all instances of the behaviour shown in (*a*–*d*). The black line is the phase-averaged curve (via (E 1)). (*g*) Comparison between the phase-averaged curves for seven different locomotion gaits. Line colours are proportional to the mean gait frequency. (*h*) Locomotion gaits from figure 7*c*, colour-coded by mean frequency. The colour scale here is the same as in (*g*). (*i*) Three-dimensional plots of the phase-averaged trajectories for five different behaviours. The first three postural modes are plotted here. (*j*) Regions corresponding to the orbits shown in (*i*) (coded by colour).

Periodic orbits in postural movements are suggestive of underlying low-dimensional dynamic attractors that produce stable behavioural templates [35]. These types of motifs have been hypothesized to form the basis for neural and mechanical control of legged locomotion at fast time scales [36]. Because our behavioural mapping algorithm is based upon similarities between postural frequencies exhibited at different times, a potential hypothesis is that pauses in behavioural space correspond to periodic trajectories in the space of postural movements (equation (3.1)). In our data, a fast running gait (the bottom-most region of figure 10*h*) corresponds to periodic oscillations of the postural time series with a clear peak at 12.9 Hz in the power spectral density (figure 10*a*,*b*). This frequency is in good agreement with previous measurements of the fly walking gait [37,38].

To systematically investigate the periodicity of the postural dynamics, for each behavioural bout, we map time onto a phase variable, a cyclic coordinate defined on the unit circle. This process is usually referred to as phase reconstruction. The method we use, Phaser [39], performs Hilbert transforms to construct phase estimations from several time series separately, then combines these estimates via a maximum-likelihood estimate that uses Fourier-series-based corrections. Here, we apply Phaser to the postural mode time series, *y_k_*(*t*), treating the correlated motions along all 50 postural eigenmodes as synchronized oscillators. We performed this reconstruction for each multi-cycle behavioural bout. After reconstructing the phases for all of the 5483 bouts of fast running observed in male flies, we observe a clear periodic pattern across several of the postural modes (figure 10*c*–*f*).

This type of analysis also brings additional insight into the subtle distinctions between our observed behavioural states. If we construct phase-averaged orbits for seven of the running behaviours, we observe many differences in the gait dynamics (see appendix E, figure 10*g*). For instance, we observe an increase in many mode amplitudes as the gait frequency increases (e.g. in modes 3, 12 and 13), as noted in previous work [40]. In addition, we also see subtle changes in phase (e.g. in mode 4), as well as a near-elimination of a period-doubled trajectory (seen in mode 14). This type of observation could allow for a more thorough understanding of speed control and gait transitions in hexapod locomotion.

We also find oscillatory postural dynamics for other stereotyped behaviours, with many behaviours resulting in a periodic orbit in postural space (figure 10*i*). These behaviours are found in many regions of behavioural space, suggesting that much of behaviour is indeed confined to low-dimensional postural dynamics. It is important to note that periodic trajectories emerge directly from our analysis, even though the wavelet transform used to define our feature vectors does not preserve phase information.

### 4.4. Differences in behaviour between males and females

**Figure 11.**
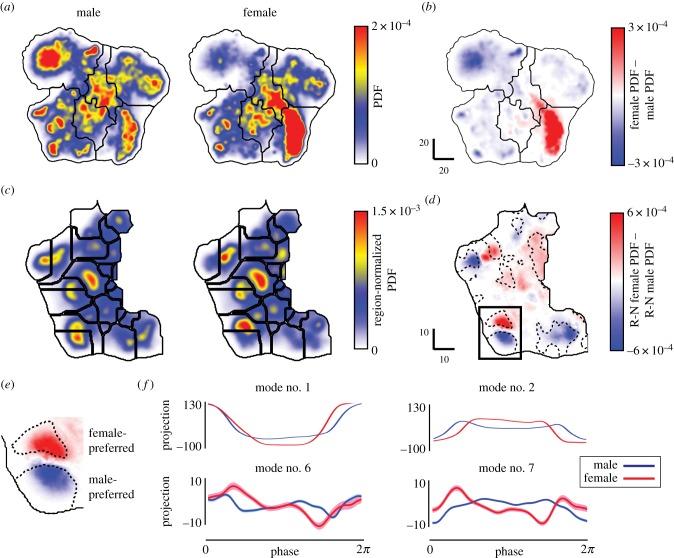
Comparison between male and female behaviours. (*a*) Measured behavioural space PDF for male (left) and female (right) flies. (*b*) Difference between the two PDFs in (*a*). Here, we observe large dimorphisms between the sexes, particularly in the ‘locomotion gaits’ and ‘idle and slow movements’ regions. (*c*) PDFs for behaviours in the ‘wing movements’ portion of the behavioural space (the lower left of the full space). These PDFs (male on the left and female on the right) are normalized so that they each integrate to one. The black lines are the boundaries found from a watershed transform and are included to guide the eye. (*d*) Difference between the two normalized behavioural spaces in (*c*). Dashed lines enclose regions in which the median male and the median female PDF values are statistically different via the Wilcoxon rank sum test (*p* < 0.01). (*e*) Zoom-in on the boxed region in (*d*). Both of these regions correspond to left-wing grooming, but with behaviours within the male-preferred region incorporating an additional leg kick (electronic supplementary material, movies S12 and S13). (*f*) Average periodic orbits for postural eigenmodes 1, 2, 6 and 7. The area surrounding the lines represents the standard error of the mean at each point along the trajectory. Average periodic orbits for all of the first 25 postural modes are shown in the electronic supplementary material, figure S6.

To demonstrate the power of this method to detect subtle differences in behaviour, we compared the behavioural spaces of male and female fruit flies by embedding the postural time-series data from females into the behavioural space derived from the male flies (figure 4). Figure 11*a* displays the male and female behavioural probability densities. We find a striking difference between the two sexes, with locomotory behaviours greatly enhanced but resting and slow motions largely suppressed in females when compared with males. This is in agreement with previous results, showing that young females are more active than their male counterparts [41].

We then sought to isolate subtle behavioural differences between the sexes that are evident in the fine-scale structure of these maps. An example of this can be seen in the ‘wing movements’ portion of the behavioural space (the lower left corner of the map). First, we obtained both male and female region-normalized (R-N) probability density functions (PDFs) (figure 11*c*), where the integral of the behavioural space density within the ‘wing movements’ region integrates to one. Within the space of wing movements, we identified regions that show statistically significant differences between the two sexes using a Wilcoxon rank sum test [42] at each point in behavioural space. This test determines the locations of significant difference between the median male PDF value and the median female PDF value (*p*-value < 0.01). Regions where significant differences were found are indicated by the dashed lines in figure 11*d*.

Particular behaviours, such as left-wing grooming, are sexually dimorphic (figure 11*d*, solid box; electronic supplementary material, movies S12 and S13). Male-preferred grooming includes a kick of the middle leg on the left side of the body that clears the profile of the wing and moves anteriorly before pushing back towards the posterior. Female-preferred grooming lacks this additional leg movement. We verified these differences by isolating the mean postural-space orbits associated with each of these regions (figure 11*f*; electronic supplementary material, figure S6). Importantly, while these orbits are statistically different, the average frequencies for the behaviours are not (*f*_male_ = 3.49 ± 0.15 Hz versus *f*_female_ = 3.28 ± 0.08 Hz). We note that these results are consistent across a large range of the behavioural-map smoothing parameter *σ* (electronic supplementary material, figure S5), such that fine-tuning of the spatial structure of the behavioural map is not necessary to obtain the results seen here.

It should be noted that future study is necessary to determine the ethological relevance of these findings and to understand how much of the variance we observe is related to the specifics of our experimental paradigm. However, the fact that these distinctions are found without specifically looking for any of them—emerging only from underlying statistics of the behavioural map—provides quantitative verification that the classifications we make are meaningful. Inherent in any unsupervised classification method is the question of how to validate its accuracy. Here, there is no ground truth with which to compare, since a significant aim of our work is to dispense with *a priori* behavioural definitions. However, by showing that meaningful distinctions and agglomerations can be made between different behavioural instances, we provide evidence that the approach introduced here can become the basis undergirding a wide range of experimental investigations into the behaviour of animals.

## 5. Conclusion

The ability to map and compare the behavioural repertoire of individuals and populations of animals has applications beyond the study of terrestrial dynamics in fruit flies. Combined with tools for genetic manipulation, DNA sequencing, neural imaging and electrophysiology, the identification of subtle behavioural distinctions and patterns between groups of individuals will impact deep questions related to the interactions between genes, neurons, behaviour and evolution. In this initial study, we probed the motion of individuals in a largely featureless environment. Extensions to more complicated situations, e.g. where sensory inputs are measured and/or controlled, genes are manipulated or multiple individuals are present, are readily implemented.

Finally, we note that the only *Drosophila*-specific step in our analysis pipeline is the generation of the postural eigenmodes. Given movies of sufficient quality and length from different organisms, spectral feature vectors and behavioural spaces can be similarly generated, allowing for potential applications from worms to mice to humans and a greater understanding of how animals behave.

## Supplementary Material

Supplementary Figures and Tables

## Data accessibility

Example code for running the algorithms can be found at https://github.com/gordonberman/MotionMapper. For access to raw image data, mail to J.W.S. at shaevitz@princeton.edu.

## Appendix A. Image processing

To isolate the fly from the background, we apply Canny's method for edge detection [19], resulting in a binary image containing the edge positions. We then morphologically dilate this binary image by a 3 × 3 square in order to fill any spurious holes in the edges and proceed to fill all closed curves. This filled image is then morphologically eroded by a square of the same size, resulting in a mask. After applying this mask to the original image, we now have our segmented image.

While our tracking algorithm ensures that the fly remains within the image boundaries, the centre of the fly and the orientation within the frame vary over time. Having obtained a sequence of isolated fly images, we next register them both translationally and rotationally with respect to a template image. The template image is generated by taking a typical image of a fly and then manually ablating the wings and legs digitally.

For our first step, we rotationally align. This is achieved through finding the angle that maximizes the cross-correlation between the magnitudes of the two-dimensional polar Fourier transforms for each image and the template. Because all translation information appears in the phase of the two-dimensional Fourier transform, this rotational alignment, based only upon the magnitude of the transform, is independent of any initial translations between the images. Accordingly, once rotational alignment is achieved, we can subsequently register the images translationally via a cross-correlation.

## Appendix B. Postural decomposition from images

The aim of the postural decomposition is to take our set of 200 × 200 aligned images and create a lower-dimensional representation that can be made into time series. Naively, one would simply perform PCA on the images, using each pixel value as a separate dimension. The fly images, however, contain too many pixels to analyse due to memory limitations.

To make this problem more tractable, we analyse only the subset of these pixels which have non-negligible variance. Many pixels within the fly image are either always zero or always saturated, thus containing almost no dynamical information. Accordingly, we would like to use only a subsample of these measurements. The most obvious manner to go about this is to find the pixels containing the highest variance and keep only those above a certain threshold. The primary difficulty here, however, is that there is not an obvious truncation point (electronic supplementary material, figure S2A). This is most likely the result of the fact that the fly legs can potentially occupy the majority of the pixels in the image but only are present in a relatively small number in any given frame. Hence, many of these periphery pixels all have similarly moderate standard deviations, making them difficult to differentiate.

A more compact scheme is to represent the images in Radon-transform space, which more sparsely parameterizes lines such as legs or wing veins. After Radon transformation, the PDF of pixel-value standard deviations has a clear minimum and we keep pixels whose standard deviation is larger than this value (electronic supplementary material, figure S2B). This results in keeping 6763 pixels out of 18 090, retaining approximately 95% of the total variation in the images. If there are *N* images in our sample, we can represent our dataset, **X**, as an *N* × 6763-element matrix. We then proceed to calculate the principal directions of variation in these data using PCA, as seen in figure 3.

Lastly, the question remains of how many modes to keep in our analysis, a task made more ambiguous due to the smoothness of the eigenvalue spectrum. Our approach to determining the truncation point is to compare the PCA eigenvalues with a null model based on the noise properties of our dataset. Specifically, we assume that the noise is due to finite data collection. Although additional errors in image segmentation and registration assuredly exist in our dataset, this set null model provides an upper bound on the number of statistically meaningful eigenmodes.

To calculate this truncation point, we take our data matrix, **X**, and shuffle each of its columns independently from one another, hence eliminating all meaningful correlations between them. Given finite sampling (even if very large), however, there will still remain some residual correlations, resulting in off-diagonal non-zero terms in the covariance matrix. Hence, if we diagonalize this new covariance matrix, the largest eigenvalue provides a resolution limit for our ability to distinguish signal from finite sampling noise. Performing this analysis, we find that only 50 modes have eigenvalues larger than this largest shuffled eigenvalue. These 50 modes account for slightly more than 93% of the observed variation in the data.

## Appendix C. Wavelet calculations

We use the Morlet continuous wavelet transform to provide a multiple time-scale representation of our postural mode dynamics. More explicitly, we calculate this transform, *W_s_*_,*τ*_[*y*(*t*)], via

C1

with

C2

Here, *y_i_*(*t*) is a postural time series, *s* is the time scale of interest, *τ* is a point in time and *ω*_0_ is a non-dimensional parameter (set to 5 here).

The Morlet wavelet has the additional property that the time scale, *s*, is related to the Fourier frequency, *f*, by

C3

This can be derived by maximizing the response to a pure sine wave, *A*(*s*, *f*) ≡ *|W_s_*_,*τ*_[e^2*πift*^]*|*, with respect to *s*.

However, *A*(*s*, *ω*) is disproportionally large when responding to pure sine waves of lower frequencies. To correct for this, we find a scalar function *C*(*s*) such that

C4

where *ω** is 2*π* times the Fourier frequency found in equation (C 3). For a Morlet wavelet, this function is

C5

Accordingly, we can define our power spectrum, *S*(*k*, *f*; *t*), via

C6

Last, we use a dyadically spaced set of frequencies between *f*_min_ = 1 Hz and the Nyquist frequency (*f*_max_ = 50 Hz) via

C7

for *i* = 1, 2, … , *N_f_* (and their corresponding scales via equation (C 3)). This creates a wavelet spectrogram that is resolved at multiple time scales for each of the first 50 postural modes.

## Appendix D. *t*-distributed stochastic neighbour embedding implementation

For our initial embedding using *t*-SNE, we largely follow the method introduced in [18], minimizing the cost function

D1

where *p_ij_* = 1/2(*p_j|i_* + *p_i|j_*),

D2

and Δ*_ij_* is the Euclidean distance between points *i* and *j* in the embedded space. The cost function is optimized through a gradient descent procedure that is preceded by an early exaggeration period, allowing for the system to more readily escape local minima.

The memory complexity of this algorithm prevents the practical number of points from exceeding ≈35 000. Although improving this number is the subject of current research [43], our solution here is to generate an embedding using a selection of roughly 600 data points from each of the 59 individuals observed (out of ≈360 000 data points per individual). To ensure that these points create a representative sample, we perform *t*-SNE on 20 000 randomly selected data points from each individual. This embedding is then used to estimate a probability density by convolving each point with a two-dimensional Gaussian whose width is equal to the distance from the point to its *N*_embed_ = 10 nearest neighbours. This space is segmented by applying a watershed transform [34] to the inverse of the PDF, creating a set of regions. Finally, points are grouped by the region to which they belong and the number of points selected out of each region is proportional to the integral over the PDF in that region. This is performed for all datasets, yielding a total of 35 000 data points in the training set.

Given the embedding resulting from applying *t*-SNE to our training set, we wish to embed additional points into our behavioural space by comparing each with the training set individually. Mathematically, let *X* be the set of all feature vectors in the training set, *X*′ be their associated embeddings via *t*-SNE, *z* be a new feature vector that we would like to embed according to the mapping between *X* and *X*′, and *ζ* be the embedding of *z* that we would like to determine.

As with the *t*-SNE cost function, we will embed *z* by enforcing that its transition probabilities in the two spaces are as similar as possible. Like before, the transitions in the full space, *p_j|z_*, are given by

D3

where *d*(*z*, *j*) is the Kullback–Leibler divergence between *z* and , and *σ*_*z*_ is once again found by constraining the entropy of the condition transition probability distribution, using the same parameters as for the *t*-SNE embedding. Similarly, the transition probabilities in the embedded space are given by

D4

where Δ*_*ζ*_*_,*x’*_ is the Euclidean distance between *ζ* and .

For each *z*, we then seek the  that minimizes the Kullback–Leibler divergence between the transition probability distributions in the two spaces

D5

D6

As before, this is a non-convex function, leading to potential complexities in performing our desired optimization. However, if we start a local optimization (using the Nelder–Mead simplex algorithm [44,45]) from a weighted average of points, *ζ*_0_, where

D7

this point is almost always within the basin of attraction of the global minimum. To ensure that this is true in all cases, however, we also perform the same minimization procedure, but starting from the point *y*(*x**), where

D8

This returned a better solution approximately 5% of the time.

Because this embedding can be calculated independently for each value of *z*, the algorithm scales linearly with the number of points. We also make use of the fact that this algorithm is embarrassingly parallelizable. Moreover, because we have set our transition entropy, *H*, to be equal to 5, there are rarely more than 50 points to which a given *z* has a non-zero transition probability. Accordingly, we can speed up our cost function evaluation considerably by only allowing *p_x|z_* > 0 for the nearest 200 points to *z* in the original space.

Lastly, we find the space of behaviours for the female datasets by embedding these data into the space created with the male training set. We find that the median re-embedding cost (equation D 5) for the female cost is only 1% more than the median re-embedding cost for the male data (5.08 bits versus 5.12 bits) indicating that the embedding works well for both sexes.

## Appendix E. Phase-averaged orbits

After applying the Phaser algorithm, we find the phase-averaged orbit via a von Mises distribution weighted average. More precisely, we construct the average orbit for eigenmode *k*, *μ*^(*k*)^(*ϕ*) via

E1

where  is the projection onto the *k*th eigenmode at time point *t_i_*, *ϕ*_*i*_ is the phase associated with the same time point and *κ* is related to the standard deviation of the von Mises distribution (, where *I*_*ν*_(*x*) is the modified Bessel function of *ν*th order). Here, we find the value of *κ* ≈ 50.3, which is the *κ* resulting in *σ*_vM_ = 0.1.

Because phase reconstruction only is unique up to an additive constant, to compare phase-averaged curves of different behavioural bouts, an additional alignment needs to occur. This is performed by first finding the maximum value of cross-correlation between the phase-averaged curves for each mode. Then, the phase offset between that pair of 50-dimensional orbits is given by the median of these found phase shifts.
