# Supplementary material for: Mapping the stereotyped behaviour of freely moving fruit flies
Source: J R Soc Interface. 2014 Oct 6;11(99):20140672. doi: 10.1098/rsif.2014.0672 (PMC4233753; doi:10.1098/rsif.2014.0672)
Supplement: Supplementary Figures and Tables [file rsif20140672supp1.pdf]

Mapping the stereotyped behaviour of freely-behaving fruit flies: Supplementary Materials

Gordon J. Berman,[1] Daniel M. Choi,[2] William Bialek,[1] and Joshua W. Shaevitz[1]

[1] *Joseph Henry Laboratories of Physics and Lewis-Sigler Institute for Integrative Genomics*

[2] *Department of Molecular Biology*

Princeton University

Princeton, NJ 08544

TABLE S1. Parameters used in eigen-decomposition

Parameter	Description	Value
N_θ	Number of angles used in Radon transforms	90
M	Number of postural eigenmodes (found, not defined *a priori*)	50

TABLE S2. Parameters used in wavelet analysis

Parameter	Description	Value
N_f	Number of frequency channels	25
ω_0	Non-dimensional Morlet wavelet parameter	5
f_{min}	High-pass frequency cut-off	1 Hz
f_{max}	Low-pass frequency cut-off	50 Hz

TABLE S3. Parameters used in t-SNE implementation

Parameter	Description	Value
H	Transition entropy	5
N_{train}	Training set size	35,000
$N_{neighbors}$	Maximum non-zero re-embedding transitions	200
N_{embed}	Nearest neighbors for training set calculation	10

TABLE S4. Parameters used in behavioural segmentation

Parameter	Description	Value
σ	Width of gaussian kernel density estimator for embedded points	1.5
τ_{min}	Minimum behavioural time scale	.05 s
σ_v	Width of gaussian for embedded-space velocity calculations	.02 s
σ_{vM}	Standard deviation of von Mises smoothing for phase averaging	.1 radians

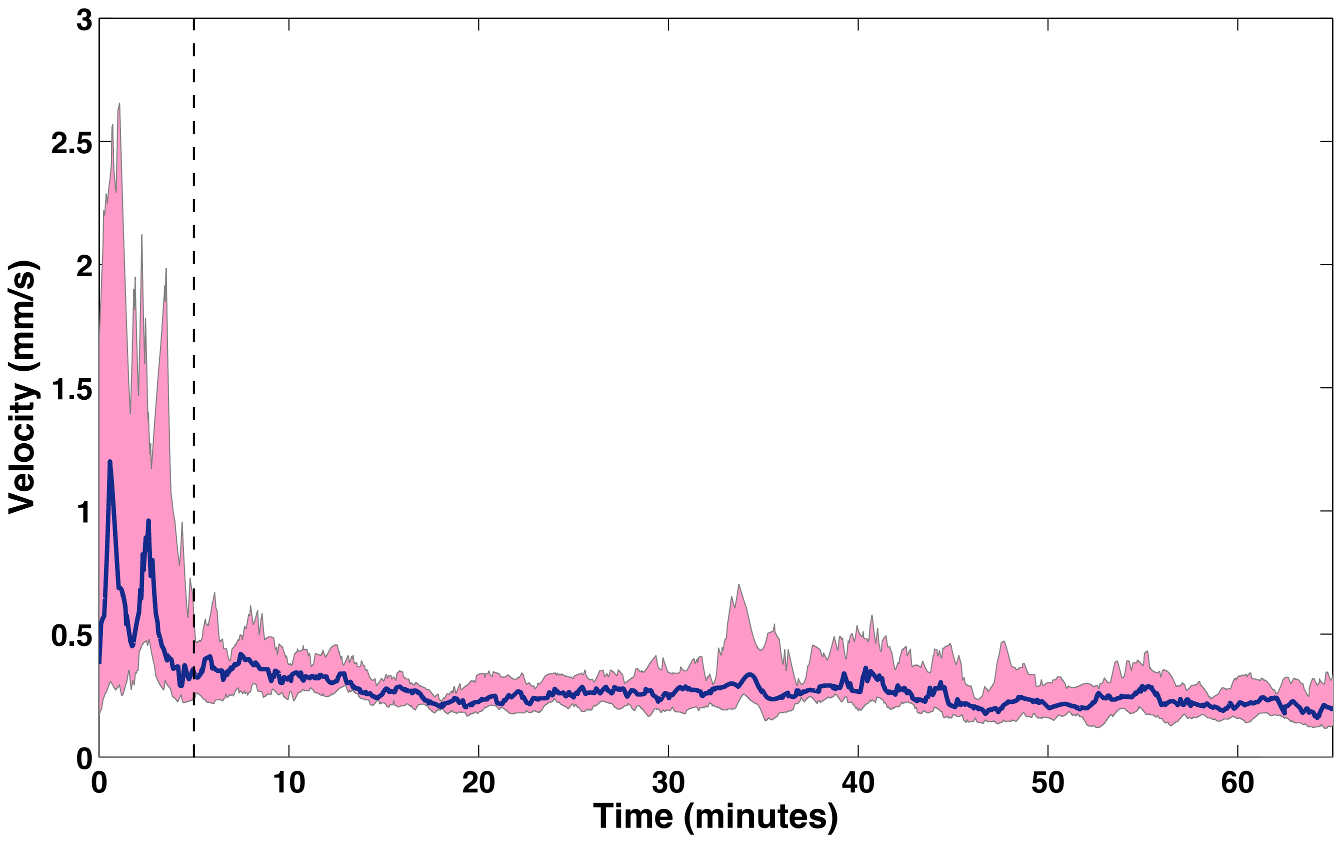

FIG. S1. Flies adapt to the arena in about five minutes. Median movement velocity for male flies ($N = 59$) during the 65 minute filming period. Pink regions represent the 25% and 75% quantiles at each point in time. Note that almost all adaptation occurs within the first five minutes placed in the dish. This region is excluded from our analyses

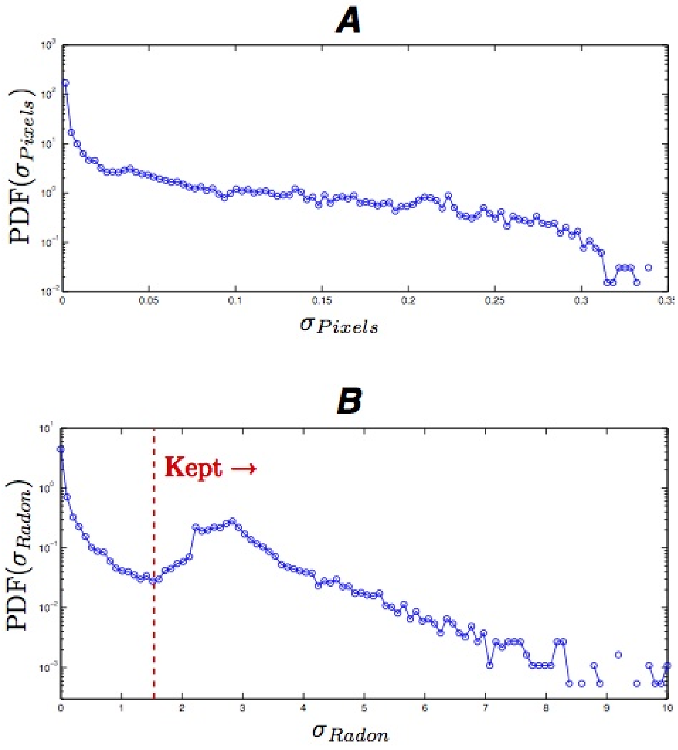

FIG. S2. Radon vs. pixel representation of images. A) Probability density function of pixel standard deviations. B) Probability density function of Radon pixel standard deviations. Note the clear minimum that exists in B), allowing for an effective reduction in the number of pixels necessary to represent the data.

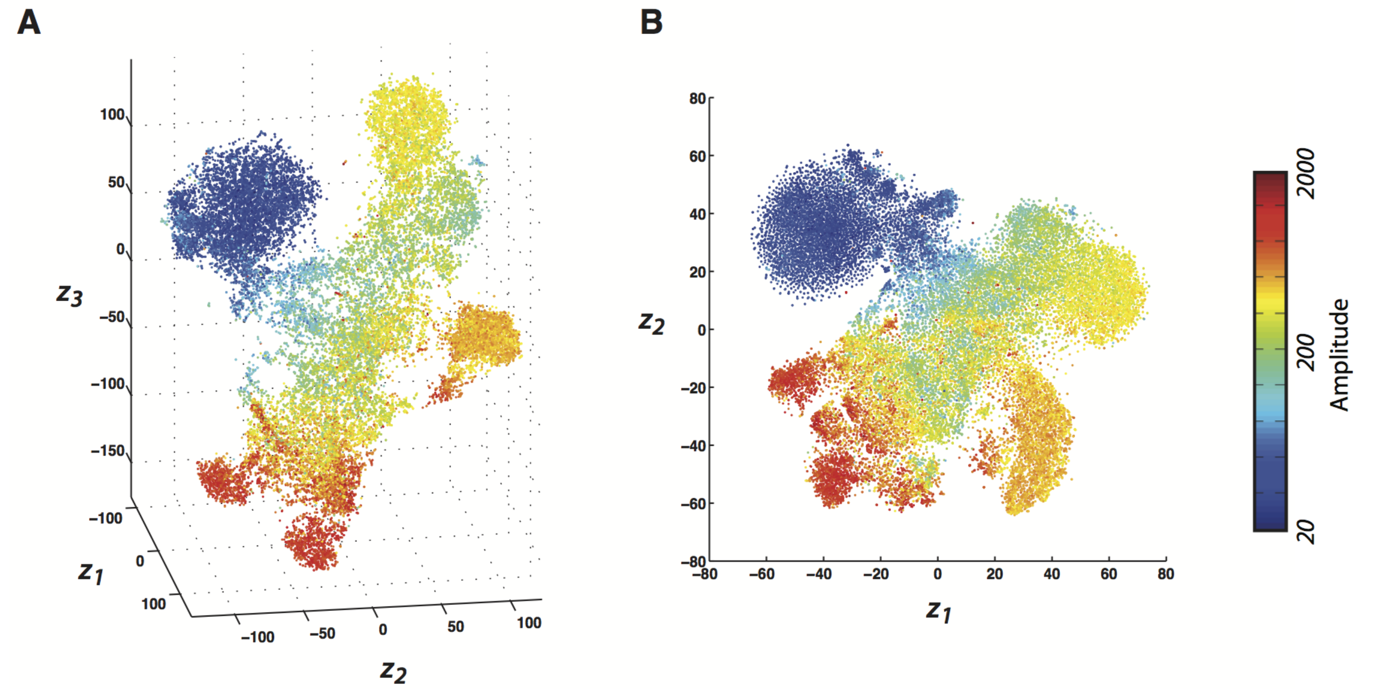

FIG. S3. Comparison between embedding into 3-D (A) versus 2-D (B) via t-SNE. Other than the embedding dimension, all other parameters remain constant. Color labels are proportional to the logarithm of the normalizing amplitude ($\sum_{k,f} S(k,f;t)$) for each point in the training set. There is a 2% improvement in the error function (Equation D1) in the 3-D case as compared to the 2-D embedding (2.9 versus 3.3 bits out of a total of 20.6 bits for the transition matrix P).

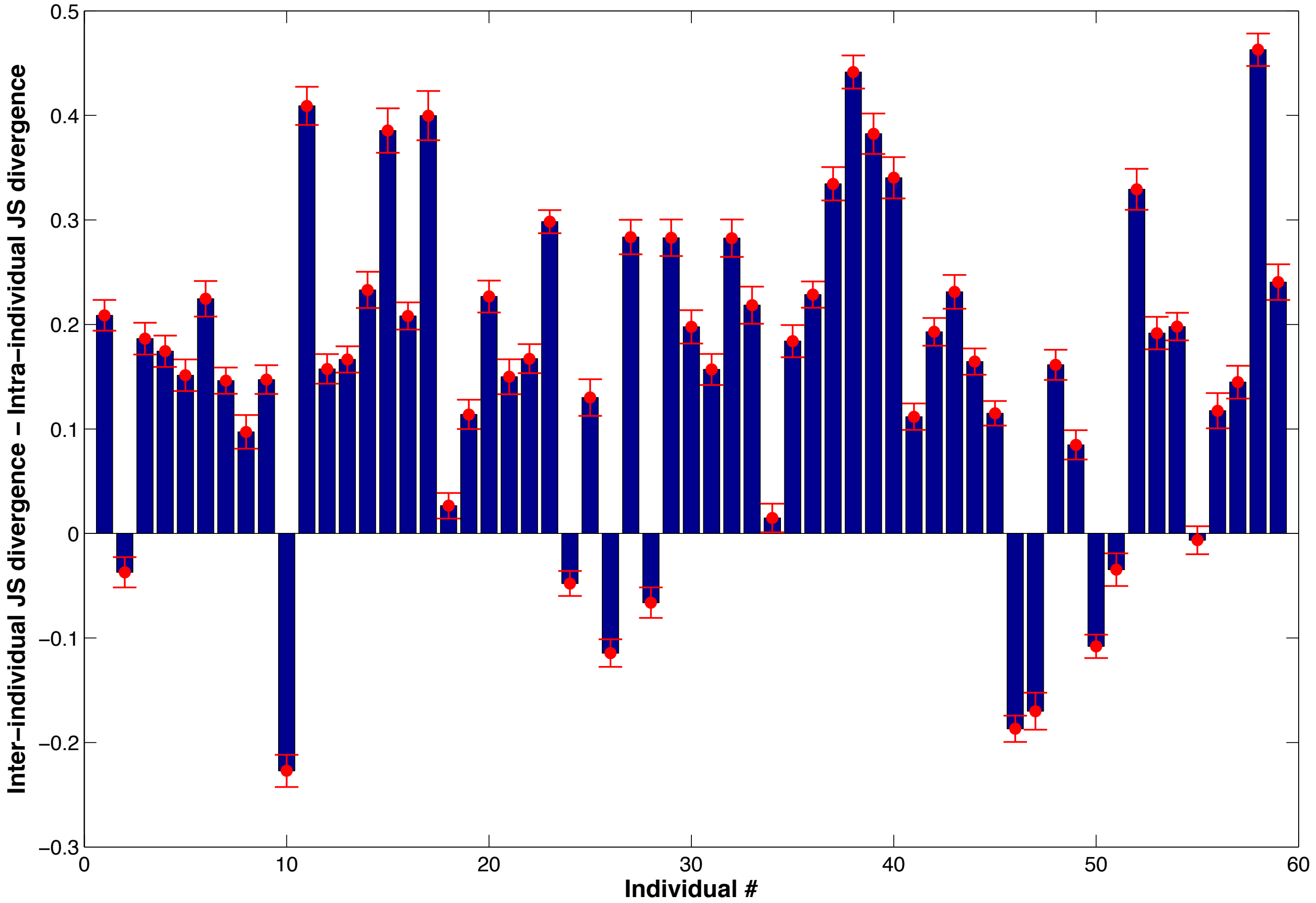

FIG. S4. Inter- versus intra- individual variation. Plotted is the inter-individual variation minus the intra-individual variation for each of the 59 male flies. Here, we measure the difference between two behavioural spaces as the Jensen-Shannon (JS) divergence between their respective probability densities. Intra-individual variation is measured as the JS divergence between maps generated from the first 20 minutes and the last 20 minutes of an individual data set. Inter-individual variation is measured as the median JS divergence between the behavioural space between individuals. Error bars are calculated via N-1 bootstrapping on the inter-individual variance. Accordingly, a positive value on this plot implies that the two portions of the data set are more similar to each other than they are to other individuals' behavioural spaces. We find that 48 of the 59 individuals display significantly less intra- than inter-individual variations ($\mu = .16 \pm .02$).

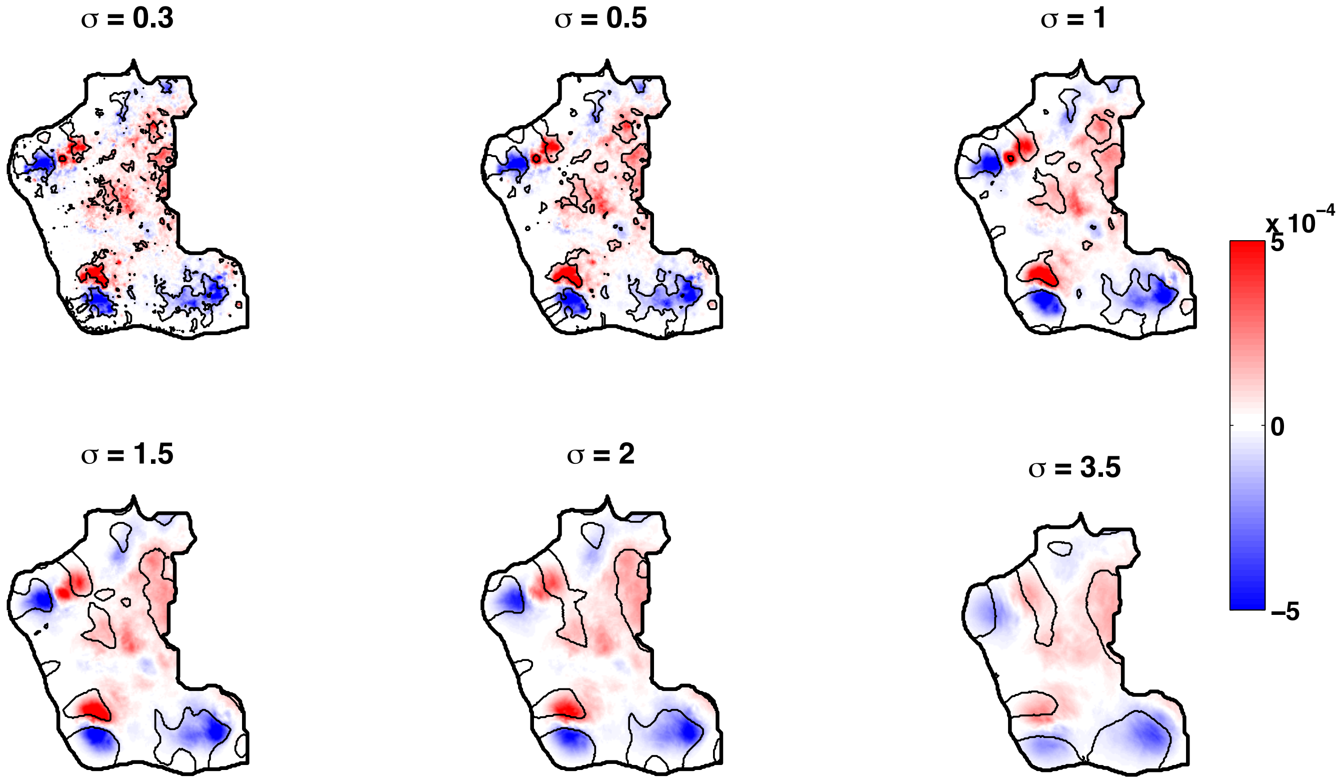

FIG. S5. Effect of varying the smoothing parameter, σ, on the identified male/female wing movement distinctions. Plots are of the difference between the median female region-normalized probability density function and the median male region-normalized probability density function for various values of σ. Lines within the regions encircle regions where the p-Value of the Wilcoxon rank sum test are less than .01.

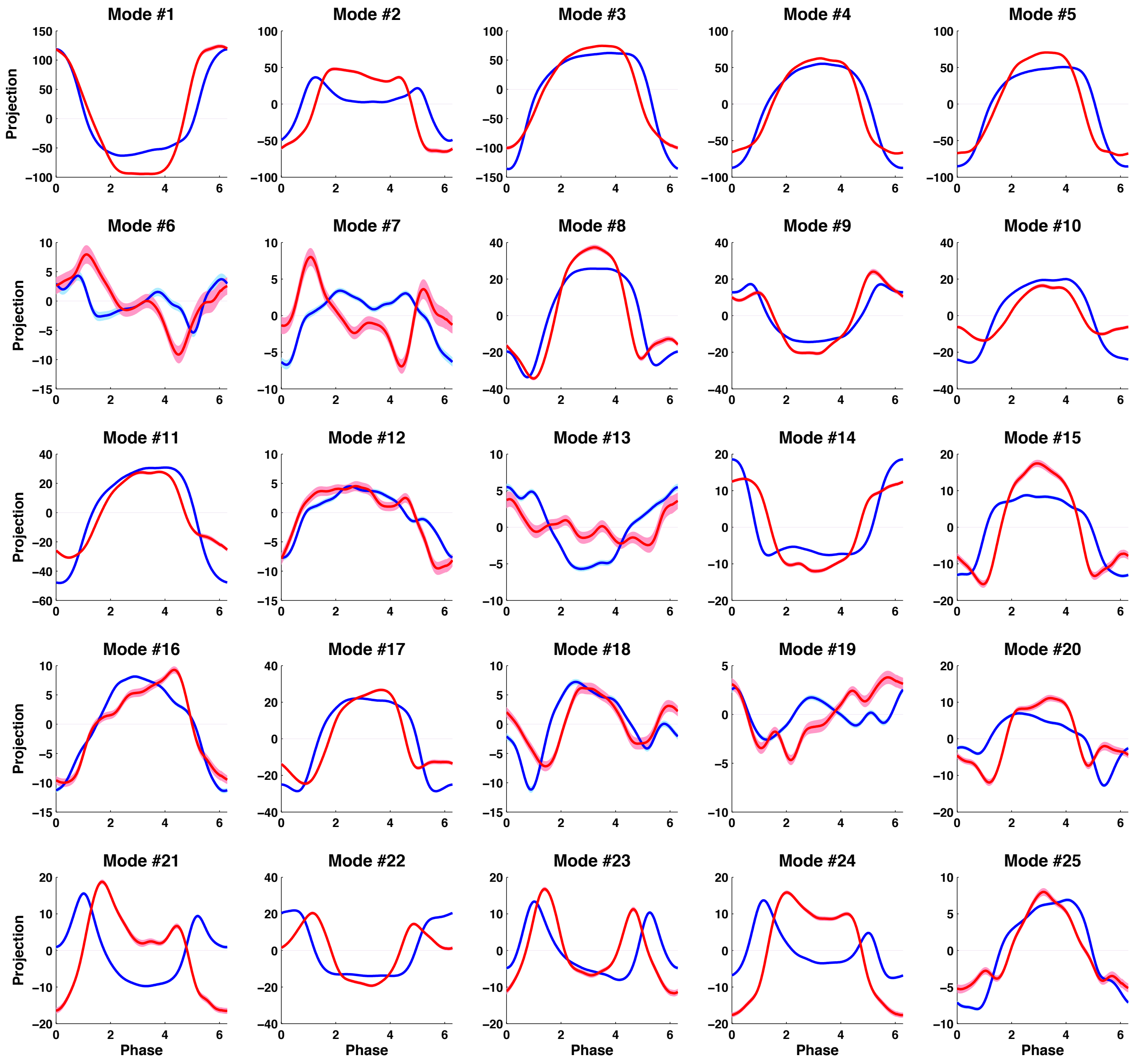

FIG. S6. Postural space periodic orbits for left wing grooming behaviours in Figure 11 in the main text. Blue curves are the average orbits for region (i), and red curves are average orbits for region (ii). Line thicknesses represent that standard error of the mean at each phase.

SUPPLEMENTARY MOVIE LEGENDS

Movie S1. Raw video data of a behaving fly (left) and the corresponding segmented and aligned data (right).

Movie S2. Dynamics in behavioural space. Raw video of a behaving *D. melanogaster* (middle) is displayed alongside coordinates of the fly's position within the filming apparatus (left) and its position in the embedded behavioural space (right). The red circles represent the positions in the appropriate coordinate system and the trailing lines are the positions traversed in the previous .5 s. The light blue shading indicates that a particular behaviour is being performed, and the blue text below the video of the fly gives a coarse label for the behaviour. The first portion of the movie is 5 s, played at real time (indicated by "Real Time" above the fly video), and the subsequent portion of the movie is slowed down by a factor of 5 for clarity (indicated by "Slowed 5×").

Movies S3-11. Each movie is a mosaic of multiple instances of specific regions in behavioural space as displayed in Fig. 8 and Table S5. Every movie contains multiple segments from many different individuals and are slowed by a factor of 4 for clarity.

TABLE S5. Behavioural Movies

**Movie**	**Label**
Movie S3	Idle
Movie S4	Right wing grooming
Movie S5	Left wing grooming
Movie S6	Left wing and legs grooming
Movie S7	Wing waggle
Movie S8	Abdomen grooming
Movie S9	Running
Movie S10	Front leg grooming
Movie S11	Head grooming

Movie S12. Composite movie (slowed by a factor of 4) of randomly chosen instances of flies from the male-preferred behavioural region in Figure 11 of the main text.

Movie S13. Composite movie (slowed by a factor of 4) of randomly chosen instances of flies from the female-preferred behavioural region in Figure 11 of the main text.
